# Morganella morganii could be an important Intensive Care Unit pathogen

**DOI:** 10.4103/0972-5229.74176

**Published:** 2010

**Authors:** Nidhi Singla, Neelam Kaistha, Neelam Gulati, Jagdish Chander

**Affiliations:** Department of Microbiology, Government Medical College Hospital, Chandigarh, (India)

Dear Editor,

Critically ill patients admitted in Intensive Care Units (ICU) are predisposed to many nosocomial infections due to underlying illnesses, various invasive procedures and prolonged hospital stay. The spread and treatment of multidrug resistant organisms, such as Methicillin Resistant *Staphylococcus aureus* (MRSA), Vancomycin Resistant Enterococci (VRE), Extended Spectrum β Lactamase (ESBL) producing organisms of family Enterobacteriaceae and Metallo β Lactamase (MBL) producing *Pseudomonas aeruginosa*, can be a very challenging task. One such pathogen, which is often ignored but is of clinical significance is, *Morganella morganii*.

*M. morganii* belongs to the tribe Proteeae of family Enterobacteriaceae. Despite its wide distribution, it was considered as an uncommon cause of infections in human beings. However, it was suggested that *M. morganii* may become an important opportunistic nosocomial pathogen in the future by William *et al* way back in 1983 when bacteremia cases due to the organism had been recorded in a cardiac surgery unit.[1] Since then, there have been various reports of this organism causing urinary tract infections, skin and soft tissue infections, meningitis and bacteremia often with fatal consequences.[2] In India, sporadic cases due to infection with *M. morganii* have been reported from time to time.[3–5] A case was reported from our own center, in which *M. morganii* was isolated from a diabetes mellitus patient with septic arthritis.[6] Another study from India has reported *M. morganii* as an important uropathogen, especially amongst indoor patients.[7]

Over a period of 6 months (January 2009-June 2009), in a mini cluster outbreak of its kind, we isolated *M. morganii* 15 times from the urine samples of 10 of ICU patients (repeated isolation four times from one patient and two times from another two patients). The male:female ratio of these 10 patients was 9:1. One patient was above 80 years of age while maximum (six patients) belonged to 40-80 years with three patients less than 40 years. The clinical diagnoses were as varied as ileal perforation, cord compression, C3-C4 fixation, occipito-parietal concussion, fracture pelvis, coronary artery disease, pulmonary fat embolism with respiratory distress, etc. However, all the patients had urinary catheters inserted and had undergone one or the other invasive procedure (four patients had undergone recent surgery and seven patients had central venous catheters inserted and were on total parenteral nutrition). Three patients presented with polymicrobial urinary infection (two with *Candida* species and one with *Klebsiella pneumoniae* along with *M. morganii*). Three patients died ultimately due to causes other than infection with *M. morganii*. No case of bacteremia due to *M. morganii* was reported. All the strains isolated had same resistogram; they were resistant to augmentin, gentamicin, amikacin, nalidixic acid, norfloxacin, cefoperazone/sulbactam, piperacillin/tazobactam, and imipenem. Only one strain was sensitive to cefoperazone/sulbactam and imipenem. All the patients were administered combination drugs along with imipenem and they responded clinically with the subsequent urine cultures showing no growth of *M. morganii*.

Although any infective site can serve as the source of bacteremia, the urinary tract accounts for the majority of cases.[8] Another study on bacteremia patients has reported urinary tract (37%) and biliary tract (22%) infections to be the major portals of entry for *Morganella* in the blood stream, suggesting the need for antibiotic coverage for *M. morganii* in debilitated patients.[9] Further, this problem is compounded by the fact that virtually all *Morganella* species are capable of producing inducible chromosomal Amp C β lactamases rendering them resistant to action of primary and extended spectrum penicillins and cephalosporins.[2] Admission of patients to the ICU or in such compromised situation warrants that organisms like *M. morganii* with proven pathogenicity could be potentially dangerous and should not be overlooked. Such infections rather need to be vigorously treated, as inappropriate antibiotic therapy combined with intrinsic resistance of *M. morganii* itself becomes an independent risk factor in high risk patients.
